# A Rare *Phaeodactylum tricornutum* Cruciform Morphotype: Culture Conditions, Transformation and Unique Fatty Acid Characteristics

**DOI:** 10.1371/journal.pone.0093922

**Published:** 2014-04-07

**Authors:** Liyan He, Xiaotian Han, Zhiming Yu

**Affiliations:** 1 Key Laboratory of Marine Ecology and Environmental Sciences, Institute of Oceanology, Chinese Academy of Sciences, Qingdao, China; 2 University of Chinese Academy of Sciences, Beijing, China; Laboratoire Arago, France

## Abstract

A rare *Phaeodactylum tricornutum* cruciform morphotype was obtained and stabilized with a proportion of more than 31.3% in L1 medium and is reported for the first time. Long-term culture and observation showed that the cruciform morphotype was capable of transforming to the oval form following the degeneration of arms by two processes. After three months of culture, four morphotypes existed in a relatively stable proportion in culture for six months (10.5% for oval, 11.3% for fusiform, 37.2% for triradiate and 41.0% for cruciform). Low temperature was particularly beneficial for cruciform cell formation. As the culture temperature decreased from 25°C to 10°C, the percentage of the cruciform morphotype increased from 39.1% to 55.3% approximately. The abundant cruciform cells endowed this strain with unique fatty acid characteristics. The strain cultured at 15°C showed both maximum content of neutral lipid in a single cell and total yield. The maximum content of fatty acid methyl esters was C16:1 for *Phaeodactylum tricornutum* cultured at four temperatures (43.82% to 50.82%), followed by C16:0 (20.47% to 22.65%). Unique fatty acid composition endowed this strain with excellent quality for biodiesel production.

## Introduction

*Phaeodactylum tricornutum* has been widely studied and reported as a model diatom [1]–[5] and is generally recognized as existing in three morphotypes: oval, fusiform and triradiate [6]–[10]. The pleiomorphic characteristics of the microalgae were first described by Douglas P. Wilson in 1946 and the organism was capable of producing four morphotypes (oval, fusiform, triradiate and cruciform) [11]. Only the first three morphotypes are common, while cruciform cells are rare and regarded as irregular. Since 1946, cruciform cells have rarely been reported. Subsequent studies on *Phaeodactylum tricornutum* have mainly concerned strains with one to three of the common morphotypes. And unlike the intensive studies on the three common *Phaeodactylum tricornutum* morphotypes, cruciform cells have been rarely reported as the difficulty of cultivation.

*Phaeodactylum tricornutum*, which is a unique diatom, aroused interest in the field of biodiesel production when the whole genome became available [2], [5], [12], [13]. However, previous studies have mainly been based on the three common morphotypes: oval, fusiform and triradiate. The differences between the three common morphotypes are first seen in cell structure. Both atomic force microscopy (AFM) and scanning electron microscopy (SEM) have been employed to investigate the ultrastructure of the three common morphotypes [7], [10]. Topographic imaging showed that the oval cells possess an outer layer of extracellular polymers and rougher surface than those of fusiform and triradiate cells and spatially resolved force-indentation curves analysis showed that cell wall composition was different in the three morphotypes, and oval cells are the only morphotype to possess silicon [10]. The girdle regions of the fusiform and ovoid forms and the arms of the triradiate form were found to be localized in organelles [10]. Morphological diversity is important in microorganisms in order to respond to changes in environmental conditions. The transformation from one morphotype to another can be triggered by variations in culture conditions such as temperature, salinity and state of media, although the effects of temperature are indistinctive [6], [7]. Hyposaline conditions can accelerate the conversion of fusiform and triradiate morphotypes to oval and round morphotypes [7]. To some extent, the relative content of the different morphotypes is a reflection of cell physiological characteristics and the environment. This is because differences in cell composition also exist between different morphotypes. Lipid and protein dry weight as well as exopolysaccharide content vary in different morphotypes [14]. The fatty acid composition of C14:0, C16:2 and C16:3 contents were significantly greater in the fusiform morphotype compared with the oval morphotype [9]. The antibacterial activity against *Staphylococcus aureus* was also different in fusiform-enriched cultures and was nearly twice that of 100% oval cells [9].With the characteristics of fast growth, easy culture, high lipid content and excellent fatty acid composition, *Phaeodactylum tricornutum* has been widely used as potential biofuel feedstocks. Lipid metabolism in microalgae is regulated by many factors such as nutrients, temperature and light [15], [16]. Temperature is a key factor which can affect cell growth and metabolic rates through nutrient absorbance, enzymatic activities and alterations in signal pathways. Previous studies have shown that the contents of eicosapentaenoic acid and polyunsaturated fatty acids were higher at lower temperatures [15], [17].

As the only representative of the suborder Phaeodactylineae, family Phaeodactylacea, genus *Phaeodactylum* [14], new insights into the polymorphism of *Phaeodactylum tricornutum*, especially the description of the forth morphotype, have important ecological significance. In this study, we successfully cultured the fourth morphotype of *Phaeodactylum tricornutum* – the cruciform morphotype in a high proportion which is reported for the first time. We also demonstrated the transformation from cruciform to other common morphotypes and clarified the fatty acid characteristics of this unique strain at different temperatures.

## Materials and Methods

### Culture conditions

The *Phaeodactylum tricornutum* strain CCMM 2004 (maintained by the Institute of Oceanography, Chinese Academy of Sciences) has been grown in f/2 medium at 18±0.5°C for several years. Sub-culturing was conducted every month to avoid possible nutrient limitation. Before this study was conducted, the particular strain was found to possess cruciform morphotype. In this study, the strain was grown in 250 mL Erlenmeyer flasks, each containing 150 mL sterilized L1 medium [18] with an initial salinity of 30 at 15±0.5°C. The light density was 4000 Lux with a 12∶12 h light: dark cycle. The initial inoculation density was appropriately 5×10^4^ cells/mL and all cultures were shaken twice a day. From January 12, 2013 to October 12, 2013, two new cultures were added each month under the same conditions. All cultures were kept without nutrients replenished.

### Microscopic observation

The morphological characteristics of *Phaeodactylum tricornutum* CCMM 2004 were observed each month using a phase contrast microscope (Olympus IX71, Japan) equipped with a DP73 digital camera. Blood counting chamber was employed for quantifying each morphotype. For each sample, approximately 1000 cells were counted for morphological statistics after mixed evenly. Samples for SEM (Hitachi S-4800, Japan) observation were prepared using a freeze dehydration procedure [19].

### PCR amplification of the 18S rDNA

For genomic DNA extraction, 10 mL cultures were collected by centrifugation at 4000 rpm using a Plant DNA Kit (Tiangen). PCR primers for 18S rDNA amplification are shown in Table 1 and were designed based on the sequences of *Phaeodactylum tricornutum* in NCBI (DQ402479, GQ452863 and HQ912556). Genomic DNA and PCR products were electrophoresed in a 1.2% (w/v) agarose gel buffered by TBE for 0.5 h before staining with ethidium bromide solution. 18S rDNA was sequenced by the Genomic Platform of IOCAS.

**Table 1:** Primers for amplification of 18S rDNA from *Phaeodactylum tricornutum* CCMM 2004.

Name	Primer Sequence (5′-3′)
18SF	AACCTGGTTGATCCTGCCAGT
18SR	GATCCTTCYGCAGGTTCACCTAC

### Growth and biomass estimation

Four temperatures (10°C, 15°C, 20°C and 25°C) were used in this study to determine the fatty acids produced by *Phaeodactylum tricornutum*. The growth of *Phaeodactylum tricornutum* cultured at different temperatures was monitored each day by reading the fluorescence value obtained by the TD700 Laboratory fluorescence spectrometer. Biomass was estimated by dry weight according to Phukan et al. [20].

### Relative content of neutral lipids in a single cell determination

For intracellular neutral lipids determination, the fluorescence dye, BODIPY 505/515, was diluted in DMSO to achieve a final concentration of 100 mM as the stock solution. 10 μL stock solution was added to 1 mL *Phaeodactylum tricornutum* cell suspension. Observations were conducted using a fluorescent microscope (Nikon Eclipse 50i, Japan) after staining for 2 min. Flow cytometry was employed to determine green fluorescence of BODIPY-stained cells, cell size and morphology and red autofluorescence caused by chlorophyll, using a BD FACSVantage SE flow cytometer (Becton Dickinson, USA) equipped with an air-cooled 488 nm argon-ion laser. The optical system in the BD FACSVantage SE flow cytometer collects green light in the FL1 channel and red light in the FL3 channel. For flow cytometry determination, microalgal solutions were diluted to a density of 1×10^4^ cells/mL before staining.

### Lipid content and composition analysis

Approximately 3 g centrifuged fresh algal pellets from each sample were collected for lipid content and composition analysis. The samples were first dried by a lyophilizer. Lipid extraction was performed using the methods of Bligh and Dyer [21] for subsequent lipid analysis. An Agilent 7890N GC/5975N MS (Agilent Technologies Inc., USA) was employed for analysis of fatty acid methyl esters (FAMEs).

### Calculation of FAME properties

Four indices including cetane number (*CN*), iodine number (*IN*), linolenic acid content and the content of FAME with ≥4 double bonds were introduced to calculate FAME properties. The equations for the former two indices were taken from Lapuerta et al [22].

Cetane number (*CN*)
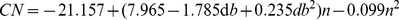
(1)


Iodine number (*IN*)
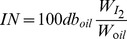
(2)


### Statistical analysis

The SAS 9.2 software system (SAS Institute Inc., Cary, NC, USA) for statistical analysis was employed in this study. One-way analysis of variance (ANOVA) was used to evaluate the differences in cell number, biomass and FAME contents following the treatments [23]. Correlation analysis was conducted using Pearson correlation coefficients. P-values <0.05 were considered significant.

## Results and Discussion

### Cell transformation between the four morphotypes

**Figure 1:**
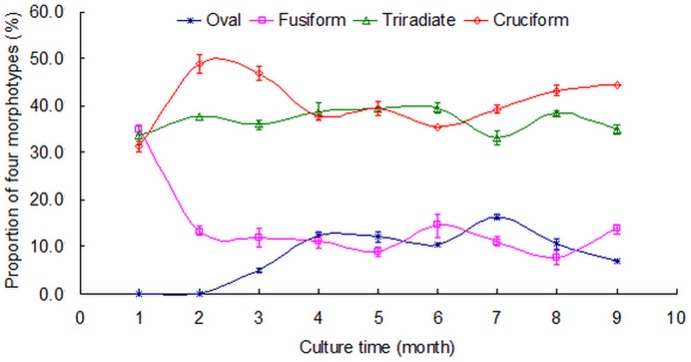
Proportions of the four morphotypes during long-term culture. Data are averages of duplicate measurements. Error bars represent standard deviation.

Following observation and counting over a nine-month period, the proportions of the four morphotypes were calculated and are shown in Fig 1. During the first two months of growth, only cruciform, triradiate and fusiform morphotypes were found in the culture. During the first month, cell numbers of these three morphotypes were similar with 31.3%, 33.7% and 34.9% of cruciform, triradiate and fusiform, respectively. After two months, the proportions of cruciform, triradiate and fusiform were 48.9%, 37.8% and 13.3%, respectively. The fusiform morphotype reduced sharply during this stage with a 21.6% decrease, and the cruciform morphotype increased by 17.6%. The oval morphotype was absent for three months. The protortion of the oval form was 5.0% of the total cell count at that time. Subsequently, the four morphotypes showed relatively stable proportions in culture during the following six months (10.5% for oval, 11.3% for fusiform, 37.2% for triradiate and 41.0% for cruciform). During the nine months' observation, proportion of triradiate cells ranged from 33.3% to 39.5% and showed a consistency compared with the other three morphotypes. While a *Phaeodactylum tricornutum* strain CCAP 1052/1A with 100% triradiate forms changed to a 50∶50 triradiate: fusiform ratio after cultured for one month and this ratio was maintained for four years [6]. Some similar researches also showed that stability of triradiate morphotype was lower than other morphotypes [24]. This study may be a special case compared with previous studies because other *Phaeodactylum tricornutum* strains often have one or no more than two preferential morphotypes in laboratory culture. Abundant morphotype compositions and complicated interactions between them may explain morphotype transformations of this strain.

**Figure 2:**
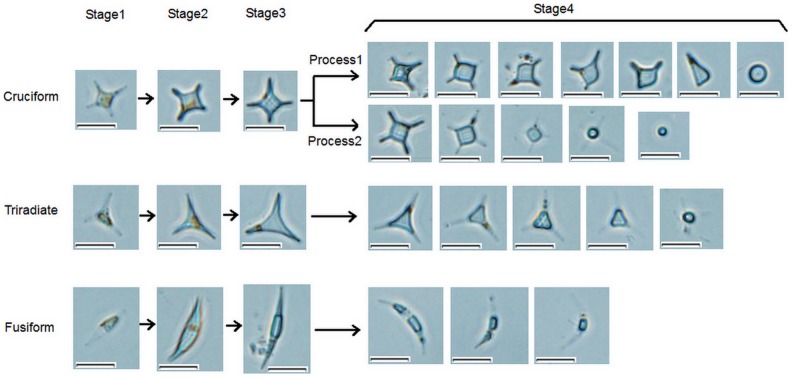
Transformation from cruciform, triradiate and fusiform to oval morphotype. This transformation involves four stages. In stage 1, the three morphotypes show the characteristics of a thin form indicating that the content of intracellular substances is poor. In stage2, the cells are plump indication many intracellular substances. In stage 3, the overall outline of the cells begins to change. In stage 4, the arms of the three morphotypes gradually disappear and the oval form is observed. Scale bars = 10 μm.

Previous studies have focused on the morphology of *Phaeodactylum tricornutum*, and the results showed three morphotypes including fusiform, triradiate and ovoid [6]–[8], [10]. In the present study another rare morphotype was detected, and the proportion of the rare cruciform morphotype was greater than 40% depending on the culture conditions (Fig. 1). Another difference between this study and previous studies was the presence of the oval form, which was not observed in this study until after three months of culture. It is likely that this morphotype was the result of the transformation of other morphotypes. Long term observation results on intermediate morphotypes may support for the above speculation. Fig. 2 shows the specific transformation processes and the changes from cruciform, triradiate and fusiform to oval. Generally, this transformation involves four stages. Stage 1 is the initial period, and the three morphotypes show the characteristics of a thin form indicating that the content of intracellular substances is poor. In stage2, the cells are plump indicating many intracellular substances. In stage 3, the overall outline of the cells begins to change. In stage 4, the arms of the three morphotypes gradually disappear and the oval form is observed. In cruciform cells, two processes related to the formation of oval cells have been found. In the first process, four arms degenerate and disappear one by one. Thus, several intermediate forms exist during this process. In the second process, four arms degenerate and disappear around the same time. For triradiate and fusiform cells, only one process has been found to result in the oval form, respectively, and this process is similar to the second process found in the cruciform morphotype.

**Figure 3:**
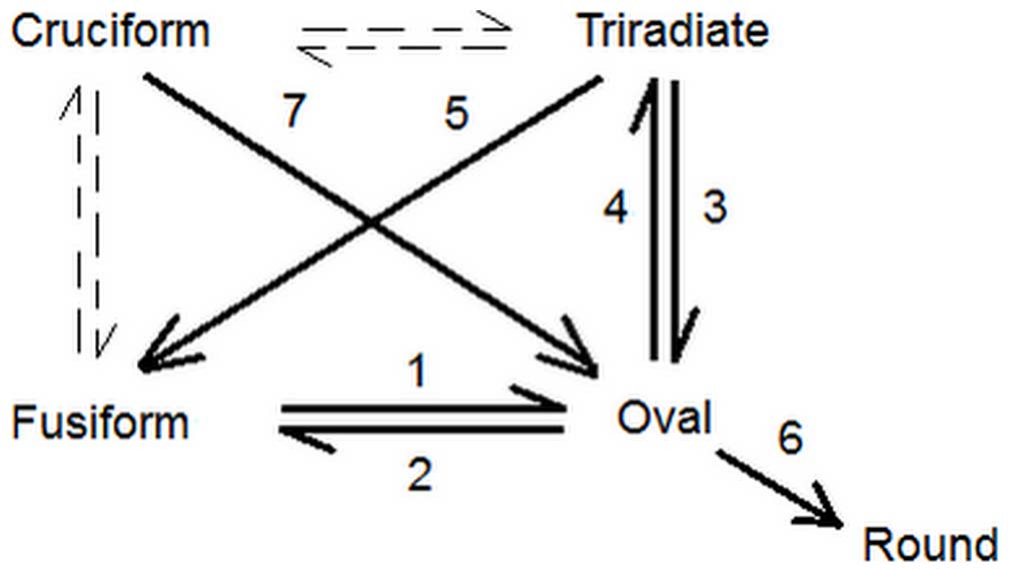
Transformations between the four morphotypes. Transformations 1–6 are study results of previous researchers. Pathway 7 observed in this study is a new discovery.

Combined with the study results of previous researchers [6], [7], [24], a new map of *Phaeodactylum tricornutum* morphotype transformation is shown in Fig. 3. Of these transformations, pathway 7 observed in this study is a new discovery. It shows that cruciform cells possess similar characteristics to fusiform and triradiate cells and degenerate arms to form oval cells.

**Figure 4:**
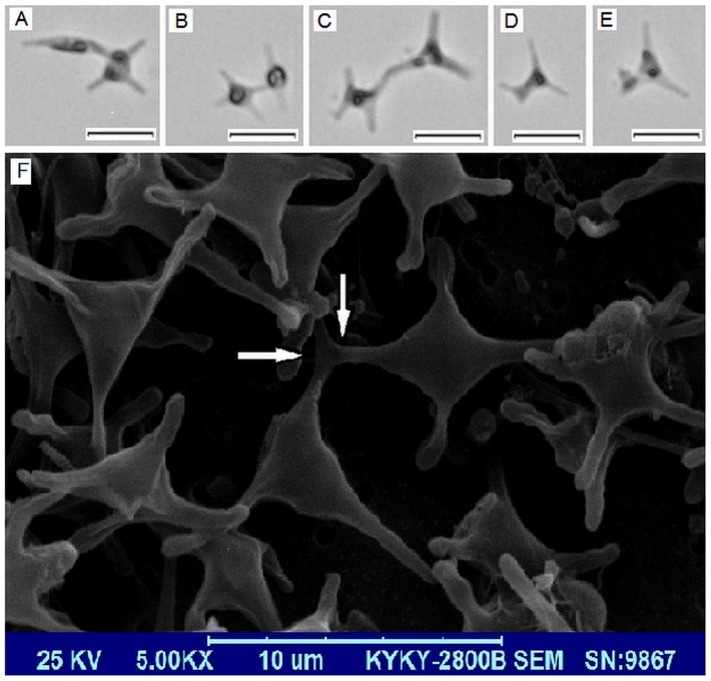
Intermediate forms in the culture. A–E are DIC photos. Scale bars = 10 μm. F is SEM result and the white arrows direct to the “conjunct arm”.

Besides the confirmed pathway, other transformations, such as cruciform to triradiate, cruciform to fusiform and the reverse transformations, may also exist. This speculation is based on several intermediate forms shown in Fig 4. Imagine A and B show two complexes of fusiform and cruciform cells with different connecting locations. Through the conjunct arm, two different morphotype cells link together. Although it is difficult to decide whether the fusiform or the cruciform pre-exist, transformations between the two forms exist. It is likely that one arm of the cruciform cell elongates and develops into a fusiform cell. A similar process may occur in triradiate and cruciform cells (Fig. 4C), because the two cells are connected by one arm. Image D and E in Fig. 4 also show the possible transformation between triradiate and cruciform cells, where a triradiate cell develops one more arm to form a cruciform cell. From the above descriptions, the “conjunct arm” plays a significant role in cell transformation. Using scanning electron microscopy, the “conjunct arm” is clearly shown in Fig. 4F.

### Sequence alignment results of 18S rDNA

After centrifugation and collection of microalgal cells, genomic DNA of *Phaeodactylum tricornutum* CCMM2004 cultured at 15°C containing all three morphotypes was extracted. Based on the template and microalgal 18S rDNA universal primers, a PCR product of approximately 1700 bp was obtained by electrophoresis. Further sequencing results showed no interference peak which indicated the purity of the strain. 1674 bp effective 18S rDNA sequences were obtained for *Phaeodactylum tricornutum* CCMM2004. The NCBI Blast nucleotide sequence showed 100% similarity with the *Phaeodactylum tricornutum* 18S rRNA sequence (GenBank accession no. CQ452863).

### Morphological characteristics of *Phaeodactylum tricornutum* cultured at different temperatures

**Figure 5:**
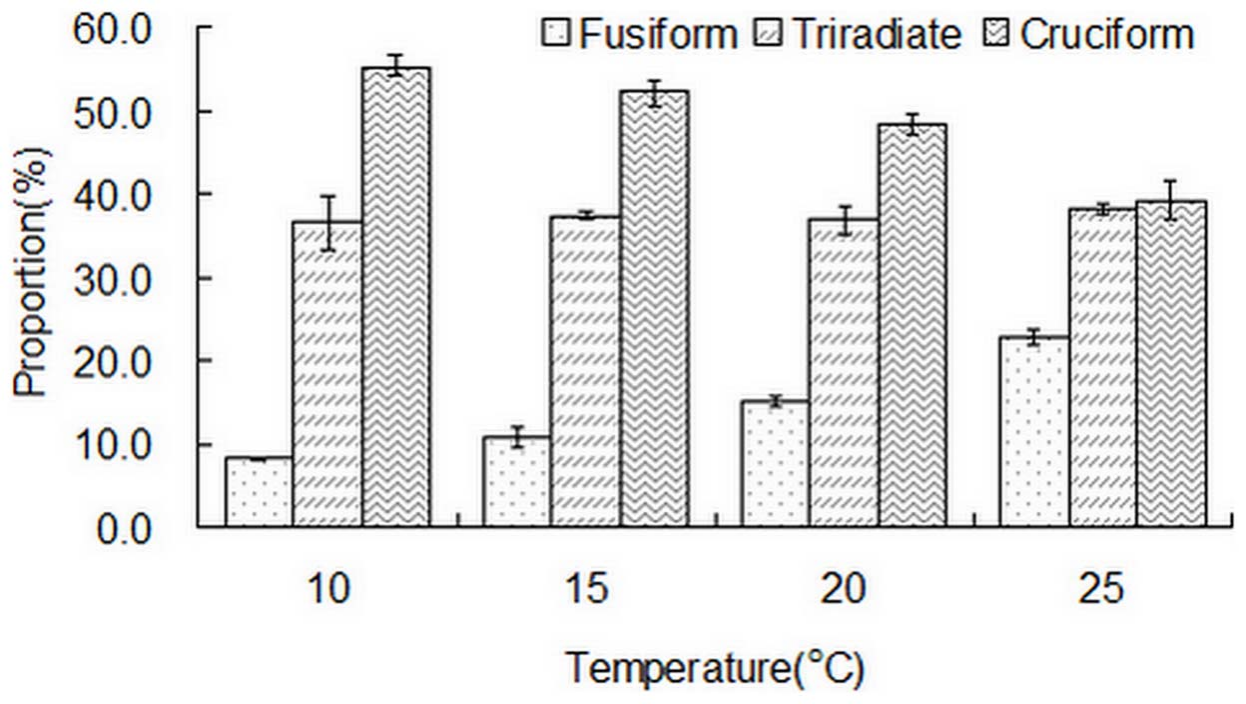
Proportion of the three morphotypes at different temperatures. Data are averages of duplicate measurements. Error bars represent standard deviation.

As shown in Fig. 5, the proportions of the three morphotypes were different depending on the culture temperature. The cruciform morphotype showed the greatest proportion and fusiform cells showed the smallest proportion. As the temperature increased from 10°C to 20°C, the percentage of cruciform cells decreased gradually from 55.3% to 48.3%, and the proportion of both fusiform and triradiate morphotypes increased. Fusiform cells increased from 8.3% to 15.0% and the triradiate morphotype increased from 36.5% to 36.7%. At 25°C, the proportions of cruciform and fusiform cells were 39.1% and 22.8%, decreasing by 9.2% and increasing by 7.8%, respectively, compared with that at 20°C. Generally, the percentage of triradiate cells showed maximum stability. Lower temperature was beneficial for cruciform cells and higher temperature facilitated the formation of fusiform cells. In this study, low temperature facilitated the formation of the cruciform morphotype. The percentage of this morphotype at 10°C was 16.2% more than that at 25°C. That may be a defence mechanism of *Phaeodactylum tricornutum* CCMM 2004 to different temperatures.

Culture temperature plays an important role in *Phaeodactylum tricornutum* morphotype characteristics. For strain CCMP633, the proportion of the oval morphotype was 60%–75% at 15°C –19°C, however, the proportion of fusiform cells was 80%–95% at 25°C –28°C [8]. Oval cell growth increased notably in strain Pt1 8.6_F_ when the culture temperature was altered from 19°C to 15°C compared to that at 28°C [7]. The proportions of the different morphotypes of *Phaeodactylum tricornutum* primarily depend on the strain itself to a great extent. Approximately 95%–100% of the *Phaeodactylum tricornutum* strains CCMP632, CCAP 1052/1A, CCAP 1052/6, CCMP630, CCMP631, CCMP1327 and MACC B228 were fusiform cells, whereas 80%–85% of *Phaeodactylum tricornutum* strain NEPCC 640 were triradiate cells [8]. When cultured in standard temperature conditions (19°C), 80% of Pt1 8.6_F_ were fusiform cells whereas 60% of Pt3_O_ were oval cells [7]. Temperature has a significant influence on the composition of microalgae. Available evidence shows that the contents and composition of FAME also varied in different morphotypes of *Phaeodactylum tricornutum* [9].

### Growth and biomass

**Figure 6:**
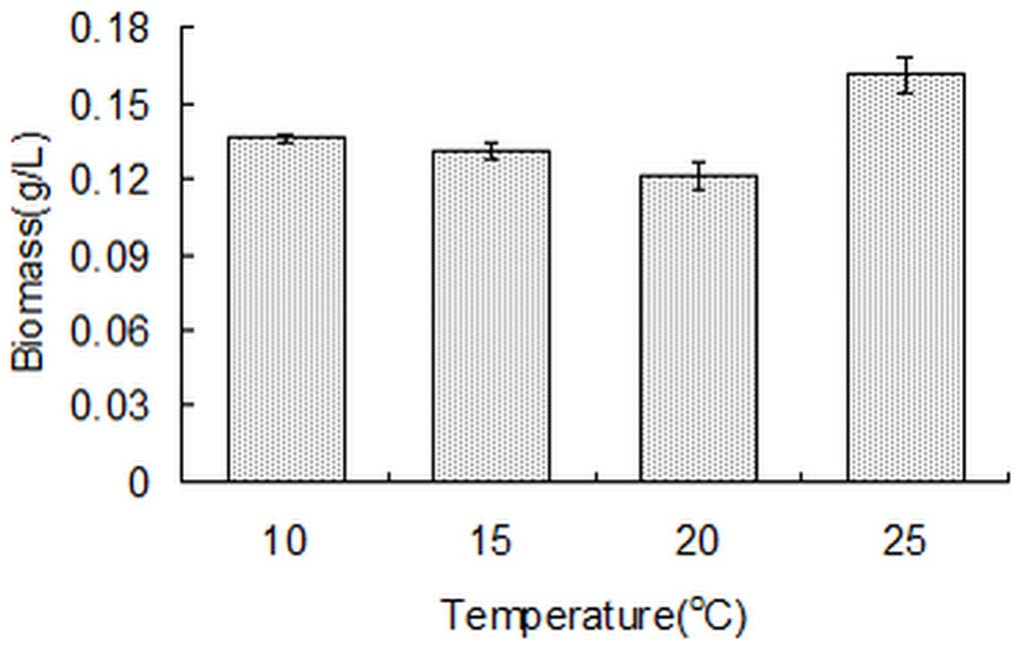
Biomass of *Phaeodactylum tricornutum* cultured at different temperatures. Data are averages of duplicate measurements. Error bars represent standard deviation.

*Phaeodactylum tricornutum* cells are capable of growing at the four temperatures studied in this report. The color of the *Phaeodactylum tricornutum* culture solution at four temperatures was also different which suggested a difference in metabolite composition and contents. The biomass yields of *Phaeodactylum tricornutum* cultured at four temperatures are shown in Fig. 6. Biomass yields at 10°C, 15°C and 20°C were about 0.121–0.136 g/L, and were less than that at 25°C (0.161 g/L). The temperature range for the growth of *Phaeodactylum tricornutum* is relatively wide [15]. This strain grows well at low temperature.

### Fluorescence staining and flow cytometry determination

**Figure 7:**
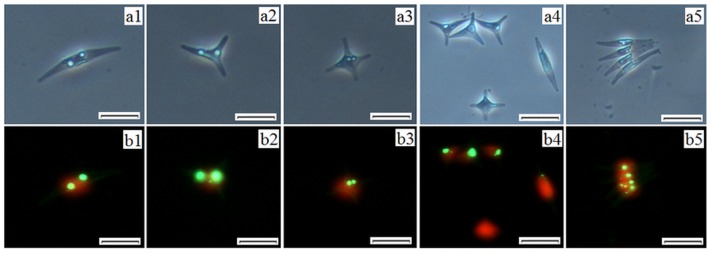
Images of *Phaeodactylum tricornutum* cells stained with BODIPY 505/515. a1, a2, a3, a4 and a5 are images at white light, while b1, b2, b3, b4 and b5 are corresponding images at the exciting light. Scale bars = 10 μm.

The refractive index of fat particles is different from that of the cytoplasm. Although the lipid dye, Nile Red, has been used for over 20 years, it has some disadvantages, which can not be overlooked. Nile Red is unable to efficiently penetrate microalgae with thick or rigid cell walls, adding to the difficulties in effective staining [25]. In addition, even for microalgae without cell walls, the stained lipids are seen as yellow and similar to chlorophyll autofluorescence red, which makes observation and quantification difficult. BODIPY 505/515 is a new fluorescence dye with a small fluorescence Stokes shift, large extinction coefficient and high fluorescence quantum yield for lipid staining [26]. It overcomes the insufficiencies of Nile Red and has been successfully used in many microalgal species [26], [27]. BODIPY-stained *Phaeodactylum tricornutum* cells showed red and specific green fluorescence when excited by blue light (Fig. 7) which is similar with previous studies on *Chlorella vulgaris* and *Chaetoceros calcitrans* [28]. The red fluorescence represents chlorophyll and the green fluorescence represents cellular lipids. Differences between them can be easily distinguished. In Fig. 7, triradiate cells showed the maximum green fluorescence intensity, followed by fusiform cells, and cruciform cells showed minimum fluorescence intensity. Different green fluorescence intensities in the three morphotypes indicated differences in lipid contents. Besides lipid contents, differences between different morphotypes of *Phaeodactylum tricornutum* also exist in other cellular characteristics. Based on the difference in extracellular polymeric substance secretion, Stanley et al. showed that oval cells were capable of adhering to both hydrophilic acid-washed glass and polydimethylsiloxane elastomer, while fusiform cells were unable to adhere to either surface [29].

*Phaeodactylum tricornutum* cells cultured at four temperatures were detected by flow cytometry. For each sample, 30000 events were carried out for statistical analysis. Distributions of cell shapes and sizes were similar in the four different treatment groups. Cells at 10°C showed more dispersion. Chlorophyll autofluorescence was reflected by FL3. The values obtained at 10°C were less than those obtained at the other three temperatures indicating less chlorophyll content in *Phaeodactylum tricornutum* at low temperature. The SSC, FSC and FL3 data showed that *Phaeodactylum tricornutum* cells grew well in the four groups which is similar with research results of Jiang et al. [15], both indicating wide temperature adaptability. After staining with BODIPY 505/515, FL1 values of *Phaeodactylum tricornutum* cells cultured at 15°C showed the greatest increase, rising from 1.04 to 151.25 (Table 1). The FL1 values of stained cells cultured at 10°C, 20°C and 25°C also showed an average increase of 72.90, 45.01 and 53.76 respectively (Table 2). These results suggest that 15°C is the optimal temperature and 20°C is relatively unfavourable for lipid accumulation in *Phaeodactylum tricornutum*. Flow cytometry can analyse the chemical composition of biomass by fluorescence labelling [30]. The combination of flow cytometry and fluorescence staining is a new method for the determination of cellular lipid. The fluorescence dyes employed for staining are primarily Nile red and BODIPY. The lipid contents of many microalgal species have been determined using this method [26], [27]. After staining with 1 μM BODIPY 505/515, the mean fluorescence value of *Chrysochromulina sp*. was 227 which was 100 times more than unstained cells [26]. The amount of fluorescence enhancement is related to the microalgal species. A fluorescence enhancement of 50–524 times in different microalgae was observed in a previous study [26], [27]. The staining efficiency of microalgal cellular lipids was thought to be related to dye concentration, staining time and cell concentration.

**Table 2:** Histogram statistical data for changes in fluorescence value.

	M1		M2		
Temperature(°C)	Mean	% Total	Mean	% Total	M1–M2
10	1.05	99.44	73.95	99.15	72.90
15	1.04	99.46	151.25	99.08	150.21
20	1.04	99.48	46.05	99.38	45.01
25	1.04	99.55	54.80	99.99	53.76

### Fatty acid content and composition

The composition and percentage of fatty acids in microalgae can be regulated by altering the culture temperature. In this study, the fatty acid profiles of *Phaeodactylum tricornutum* cultured at four temperatures are shown in Table 3. The maximum lipid contents at four temperatures were all C16:1. As the temperature increased from 10°C to 25°C, the percentage of C16:1 increased from 43.82±0.16% to 50.82±0.21%. These results are similarly to those of Jiang et al. [15] who showed that lipid contents in *Phaeodactylum tricornutum* 2038, with the exception of the percentage of C16:1, were all about 10% less than their counterpart in the present study. C16:1 content in *Phaeodactylum tricornutum* CCAP1055/1 can reach 46.35±2.3% which is the maximum content [31]. The content of C16:0 was also high at approximately 20%. The sum of C16:1 and C16:0 can reach 66.47% and was a maximum of 71.71% in this study. Saturated fatty acid contents range from 41.72% to 44.68% which is beneficial for biodiesel cetane number and oxidative stability [22]. Over 50 years ago, researchers discovered that different morphotypes of *Phaeodactylum tricornutum* differed not only in their shapes, but also in their biomacromolecule contents. Two *Phaeodactylum tricornutum* cultures dominated by fusiform and oval cells, respectively, showed different protein dry weight (41% and 34%, respectively), lipid dry weight (34% and 24%, respectively) and exopolysaccharide content (<1% and 16%, respectively) [14]. In recent years, the contents of the fatty acids, EPA, HTA and PA extracted from fusiform cells were higher than those in oval cells (42.7 and 36.6×10^−13^ g, respectively) [9]. Research on *Phaeodactylum tricornutum* 2038 showed that the percentage of PUFAs in total fatty acids decreased gradually from 31.9±1.0% to 21.8±0.4% as the culture temperature increased from 10°C to 25°C [15]. In the present study, as shown in Fig. 5, in the three growth periods, the percentage of PUFAs in total fatty acids fell to a minimum mean value of 19.04% at 10°C which was lower than that at 15°C, 20°C and 25°C (25.90%, 26.12% and 24.03%, respectively). With regard to the contribution of C16:1 and C18:1, the percentage of MUFA in total fatty acids in all treatments was maximum. Based on previous research results and those of the present study, we consider that the unique fatty acid characteristics of *Phaeodactylum tricornutum* may be caused by the presence of abundant cruciform cells.

**Table 3:** Fatty acid composition of *Phaeodactylum tricornutum* cultured at different temperatures.

	Temperature (°C)			
FAME	10	15	20	25
C8:0	4.71±0.02	5.12±0.01	4.01±0.00	4.29±0.03
C14:0	6.12±0.03	6.45±0.02	7.65±0.07	7.47±0.05
C16:1	43.82±0.16	45.86±0.09	47.34±0.17	50.82±0.21
C16:0	22.65±0.09	20.47±0.07	21.56±0.02	20.89±0.11
C18:3	1.79±0.02	1.66±0.00	1.04±0.01	0.90±0.02
C18:2	1.76±0.03	1.82±0.06	2.44±0.01	2.00±0.05
C18:1 (cri-9)	6.69±0.08	6.72±0.04	5.23±0.02	3.82±0.07
C18:1 (trans-9)	1.26±0.03	0.82±0.01	0.97±0.03	0.74±0.02
C18:0	2.30±0.02	2.06±0.06	1.88±0.05	1.95±0.01
C19:0	8.90±0.04	9.02±0.03	7.88±0.05	7.12±0.03
SFA	44.68±0.12	43.12±0.09	42.98±0.15	41.72±0.18
PUFA	3.55±0.07	3.48±0.09	3.48±0.02	2.90±0.04
C14-C18	86.39±0.31	85.86±0.27	88.11±0.35	88.59±0.24

Content of C16:1 was greatest in all FAMEs and it showed a significant correlation with *Phaeodactylum tricornutum* morphotypes. Specifically, the percentage of fusiform and triradiate morphotypes showed significant positive correlations (P<0.01 and P<0.05, respectively) with the content of C16:1. A significant negative correlation (P<0.01) was observed between the percentage of the cruciform morphotype and the content of C16:1. A previous study showed similar results. Research on *Phaeodactylum tricornutum* SAG1090-6 enriched oval and fusiform cells showed that C14:0, C16:2 n-4 and C16:3 n-4 contents in fusiform cells were significantly greater (*P*<0.05) than in oval cells [9].

### Characteristics of biodiesel produced from *Phaeodactylum tricornutum*

In the European Union, Standard EN 14214 was drafted for biodiesel use in vehicles. ASTM Biodiesel Standard D 6751 was also drafted in the United States. Of the indices included, the cetane number is the parameter used to evaluate diesel ignition quality. In this study, the cetane numbers for biodiesel at the four temperatures are shown in Table 4, and range from 64.3 to 65.2. These values conform to both EN 14214 (51 min) and D6751 (47 min) [32]. As a measure of total unsaturated oil, the iodine index was also employed in this study. The iodine indices at the four temperatures were below the maximum standard of 120 (EN 14214). Other indices included in the above standards involved restrictions on the fatty acid profile such as linolenic acid content, content of FAME with ≥4 double bonds and kinematics viscosity. The former indices were introduced to restrict excess double bonds which are easily oxidized. Kinematics viscosity is affected by both saturated and short-chain fatty acid contents.

**Table 4:** Biodiesel properties at the four culture temperatures.

Properties	10°C	15°C	20°C	25°C	Standard^a^
Cetane number	65.2	64.6	64.8	64.3	51 min
Iodine index	60.3	61.9	61.4	62.2	120 max
Linolenic acid content	1.79	1.66	1.04	0.90	12.0 max
Content of FAME with ≥4 double bonds	0	0	0	0	1 max

a The standard here refers to EN 14214.

## Conclusion

This is the first study to induce a *Phaeodactylum tricornutum* strain rich in the rare cruciform morphotype (maximum of 55.3±1.2% in this study). Long-term culture and observation showed that cruciform cells were capable of transforming to oval cells following the degeneration of arms by two processes: four arms degenerate and disappear one by one or around the same time. This new discovery deepens the understanding of *Phaeodactylum tricornutum* polymorphism. This study also demonstrated the unique fatty acid profiles of the strain cultured at different temperatures, and these characteristics were partially because of abundant cruciform cells. Moreover, the increase in cruciform morphotype at low temperature reflects the adaptability of this strain to environmental change. These findings provide a platform for further research on the cold-resistance mechanism of this strain.
